# Impact of Gender and Relationship Status on Young People’s Autonomy and Psychological Wellbeing

**DOI:** 10.3389/fpsyg.2020.01735

**Published:** 2020-07-29

**Authors:** Francisco Javier García-Castilla, Isabel Martínez-Sánchez, Gema Campos, Delia Arroyo Resino

**Affiliations:** ^1^Faculty of Law, Universidad Nacional de Educación a Distancia, Madrid, Spain; ^2^Faculty of Education, Universidad Nacional de Educación a Distancia, Madrid, Spain; ^3^Faculty of Education, Universidad de Alcalá de Henares, Alcalá de Henares, Spain; ^4^Faculty of Education, Universidad Internacional de La Rioja, Logroño, Spain

**Keywords:** wellbeing, young people, autonomy, relationship, transition to adulthood

## Abstract

This study uses scales of autonomy and psychological wellbeing to determine whether young people’s gender and romantic relationship status give rise to differences in relation to a series of specific dimensions. To this end, we used Ryff’s Model of Psychological Wellbeing, which comprises several dimensions: self-acceptance, positive relations with others, autonomy, environmental mastery, personal growth, and purpose in life; and our own Transition to Adulthood Autonomy Scale (EDATVA), whose dimensions are: self-organization, understanding context, critical thinking, and socio-political engagement. As a result, a quantitative study was performed with 1,148 young people aged 16–21 from Madrid, Spain and Bogotá, Colombia, of whom 60.2% were female and 39.8% were male. The findings show that in the gender variable there are differences between males and females in the dimensions of positive relations with others, personal growth (wellbeing questionnaire), and understanding context (autonomy questionnaire); the female sample obtained the highest scores. In the relationship variable, differences were found in environmental mastery and purpose in life; higher scores were obtained by young people in a romantic relationship. However, no differences were found in the different dimensions in the autonomy questionnaire between young people in a relationship and those not.

## Introduction

In contemporary society, social change happens at lightning speed and has a direct impact on the behavior and actions of cohorts of young people (De-Juanas and García-Castilla, 2018). Aspects such as relationships with others, communication channels in the digital world, the immediacy of the here-and-now, the desire to explore, the emergence of new social and influential groups, such as peers, the formation of romantic relationships, etc., converge to create a variety of models which positively or negatively affect the dimensions of young people’s autonomy and psychological wellbeing. Nowadays, young people delay their transition to adulthood due to various social, cultural, economic, family and personal variables. For example, time spent in education is extended and studies are finished later, more time is invested in leisure and wellbeing, they delay leaving home, they delay joining the labor market and, as a result, they also delay marriage and parenthood (Cohen et al., 2003; Rivera et al., 2011).

In this context, Arnett (2000) argues that the stage of *emerging adulthood* can be placed somewhere between the ages of 18 and 25. Others place the end of adolescence and the beginning of emerging adulthood between the ages of 16 and 21 (Berger, 2016; Maree and Twigge, 2016). As a result, the transition from adolescence to adulthood is extended, allowing adolescents more time to acquire experiences; develop life skills and communication abilities; adapt their social behavior to the environment, and shape their values. It is also a period in which other facets of interest can emerge owing to the fact that adolescents now have more time than any other period in their lives to individually explore the learning possibilities and relationships that life has to offer (Arnett, 2001).

During this broad period that represents emerging adulthood, important dimensions for the development of the individual, such as autonomy and psychological wellbeing, are progressively acquired and formed. This involves the development of competencies and skills in the various dimensions that constitute autonomy, along with the perceptual formation of psychological wellbeing in relation to processes of social interaction and experiences.

Consequently, this study aims to determine whether young people’s gender and romantic relationship status gives rise to differences in relation to psychological wellbeing and autonomy. To this end, we used Ryff’s Psychological Wellbeing Scale (1995) with its six dimensions (self-acceptance, positive relations with others, autonomy, environmental mastery, personal growth and purpose in life) and the Transition to Adulthood Autonomy Scale (EDATVA) created by Bernal et al. (2019) (self-organization, understanding context, critical thinking and socio-political engagement). A quantitative study was performed with 1,148 young people from Madrid, Spain and Bogotá, Colombia aged 16–21.

## Young People’s Psychological Wellbeing and Autonomy

Before we begin referencing other authors with the aim of establishing plausible explanations for the results in this study – which considers psychological wellbeing and autonomy as principal variables in determining differences by gender and relationship status – it is important to define what we understand by transition to adulthood. Transition to adulthood is a major developmental period in a young person’s life that entails leaving childhood behind and moving toward adulthood. This involves undergoing a series of processes that require the individual to acquire social competences and skills to help them lead an autonomous and independent life in society. In this regard, emancipation is paramount, as it consists of processes that facilitate the acquisition of competences relating to psychological wellbeing in areas such as health, education, training, employment, leisure and relationships (family, friends, partner, at school, in social environments, etc.). Both autonomy and psychological wellbeing help to protect young people from negative emotional situations that may arise during their transition to adulthood (Reis et al., 2000; Inguglia et al., 2014).

### Young People’s Psychological Wellbeing

Maturation in young people involves fundamental life-based learning associated with achieving goals and planning itineraries that lead to successes and/or failures. The results of this journey are related to young people’s values, their ability to cope, make decision, harness strengths, their self-realization, and whether they think about family and/or the collective wellbeing (Deci and Ryan, 2008; Waterman et al., 2010; Adler and Seligman, 2016).

For achievements to permeate young people’s perception of their psychological wellbeing, they must target their personal development toward attaining life skills (García-Castilla et al., 2016) that give rise to positive assessments of happiness (Melo and Mota, 2013); peer acceptance (Arnett, 2015; Campione-Barr et al., 2015; Oudekerk et al., 2015; Jorgensen and Nelson, 2018); image or physical appearance and self-esteem (Rocha, 2008); capacity to undertake challenges – and risks – in relation to personal growth and social relationships, based on the quality of social and personal interaction such as establishing romantic relationships (Melo and Mota, 2013; Hausler et al., 2017) or living as a couple. The global outcome depends on young people’s level of self-esteem and the assessment they make of this evolutionary stage. Consequently, psychological wellbeing can be considered an important variable for the achievement of young people’s overall wellbeing.

Psychological wellbeing requires precise conceptualization, having been historically associated and assimilated with terms such as quality of life and mental health (Loera et al., 2017). Thus, while quality of life refers to both material and non-material aspects, mental health and psychological wellbeing involve intangible factors of everyday reality.

In turn, mental health uses clinical signs and symptoms in its remit, while psychological wellbeing brings together personal and social dimensions that individuals evaluate subjectively. Nevertheless, the different conceptualizations of psychological wellbeing all coincide in including aspects relating to work, family and, in this case, relationship status, as well as evaluating the frequency and intensity of the relationships and emotions experienced (Loera, op. cit., 2017).

However, the definition of wellbeing proposed by Ryff and Keyes (1995) has particular relevance as it commands a broad consensus in the scientific community (Zubieta et al., 2012). The authors argue that psychological wellbeing is a construct composed of six dimensions – self-acceptance, positive relations with others, environmental mastery, autonomy, purpose in life and personal growth – that measure the subjective perception of affect and cognition in social and family relationships (Ryff and Keyes, 1995). As mentioned earlier, this is linked to the concept of happiness and the perception of satisfaction with one’s own life, which comprises professional achievements and personal goals (De-Juanas et al., 2013).

In this study, we used the Ryff Psychological Wellbeing Scale (Ryff, 1989; Ryff and Keyes, 1995), which defines six dimensions:

– Self-acceptance: an individual’s positive or negative assessment of his or herself. It implies the recognition of one’s own strengths and weaknesses.
– Positive relations with others: the ability to establish stable social relationships, emotional intelligence being a positive sign of psychological wellbeing.
– Environmental mastery: the ability to generate favorable environments consistent with personal interests and tastes. It is related to internal locus of control and the ability to influence the environment.
– Autonomy: an individual’s ability to maintain individuality with respect to others. A high score is a positive sign of resistance to social pressure.
– Purposes in life: an individual’s ability to set long-term goals and establish ways to achieve them.
– Personal growth: an individual’s ability to implement strategies that benefit the full development of their potential.

Loera et al. (2017) highlight the importance of evaluating psychological wellbeing during adolescence, given that this population is especially vulnerable due to the physical, psychological, and cognitive changes that transpire during this transformative phase. Everything that was established and accepted up to that moment in time in the family environment is questioned, and other social groups or subsystems such as friends and partners start to have more influence.

### Young People’s Autonomy

Our aim in this study was to take a more detailed look at young people’s level of autonomy in relation to gender and relationship status. One of the reasons for this was to build on studies that correlate autonomy with psychological wellbeing in young people. Results show that the greater an individual’s level of autonomy the more positive their emotional state. Higher scores are obtained by young people who have greater autonomy in their life projects (Reis et al., 2000; Inguglia et al., 2014).

To this end, we used the Transition to Adulthood Autonomy Scale (EDATVA) (Bernal et al., 2019), which is a standardized model that quantifies level of autonomy based on the assessment of young people’s decision-making processes. It consists of four dimensions that refer to the capacity of self-organization, critical thinking, understanding context and socio-political engagement. These dimensions denote an individual’s levels of autonomy, conceived from a holistic perspective.

Autonomy is a construct that has a multitude of applications in various scientific and academic fields; each with its own conceptualization. However, there are three fundamental approaches to autonomy that are described in the following paragraph. According to Cheon et al. (2019), an individual’s need for autonomy is an inherent quality of their transition to adulthood, which increases the more they advance toward maturity. A lack of autonomy leads to feelings of frustration and dissatisfaction, which interferes with their level of academic and professional commitment and undermines their level of subjective wellbeing (Riley, 2015; Soenens et al., 2017a; Liga et al., 2018; Villarosa and Ganotice, 2018; Cheon et al., 2019).

First, from an intrasubjective perspective, autonomy is defined as an individual’s capacity or ability to decide and motivate their behavior in accordance with their own principles and criteria. This vision is linked to self-realization, exercising free will, and prioritizing their own priorities, which gives rise to feelings of wellbeing when accomplished (Schüler et al., 2016; Soenens et al., 2017b). This is especially relevant during adolescence and the transition to adulthood given that this period is influenced by the dependence on other factors that exert control over individuals (Liga et al., 2018).

Second, from an interdisciplinary perspective, autonomy is understood as a process structured by multiple variables that can be conceptualized from various paradigms.

And lastly, from an interactional approach, autonomy is the result of the dependent relationship between individuals and their context. This constitutes a continuum of progression over time as they build a relationship with their environment. In this relationship, individuals are immersed in a dichotomy of dependency and independence during the transition to adulthood, in which they gradually attain greater levels of freedom to act.

The abovementioned EDATVA scale comprises the following dimensions:

1. Self-organization can be examined from a subjective perspective. Individuals organize their time to plan the activities they will participate in, according to personal choices based on their priorities (Lammers et al., 2016). According to Negru (2016), the capacity for self-organization involves personal identity and the degrees of freedom that implies. This constitutes a complex dimension that is influenced by a multitude of aspects that condition the environment in which it develops (Riley, 2015). According to Lammers et al. (2016) all individuals experience the desire to self-manage and act with interdependence with respect to others. And it is precisely this prospect that is measured by this dimension.
2. Understanding context evaluates the dimension through which individuals interact with their context and the variables that define it. Therefore, this dual nature must be considered as a subjective and collective dimension that allows it to be analyzed from both perspectives (Oshana, 2016; Reis et al., 2018). For Stenling et al. (2015), young people find factors in their environment that contribute to their autonomy and, consequently, increase their wellbeing. Identifying those factors can be key to developing interventions to enhance young people’s empowerment (Stenling et al., 2015).
3. Critical thinking, the nature of which is eminently subjective, associates the individual’s preferences and ideals to the rights they can exercise. Critical thinking enables the individual to establish their position as regard the different situations that affect or interest them, by adopting a position to preserve their interests in each scenario (Van Petegem et al., 2015). According to Narayan (2018), despite its apparent independence, context is fundamental in framing potential decisions and aspects in which an individual’s critical capacity can be activated. Riley (2015) argues that educational style and socialization processes exercise a determining influence on the development of critical thinking and on how it is activated so that that individuals can exploit different academic and life opportunities.
4. Socio-political engagement involves the participation of the individual in relation to the group in community decision-making processes that take place in society. This dimension is connected to social life and contemporary citizens’ rights insofar as exercising those rights is made possible in a context characterized by the capacity to decide and intervene in public processes in regulated decisions (Harris, 2016; Fahmy, 2017; Young, 2017). The capacity for socio-political engagement, therefore, translates into an individual’s commitment to the society they belong to and enables them to act according to the channels provided for doing so in a given place and time (Luginbuhl et al., 2016).

## Materials and Methods

This study has two main objectives. The first, to analyze whether there are differences by gender (male and female) in the different dimensions of the autonomy questionnaire (self-organization, understanding context, critical thinking and socio-political engagement) and the wellbeing questionnaire (self-acceptance, positive relations with others, autonomy, environmental mastery, personal growth, and purpose in life). And in those dimensions where gender differences were found, to perform a study to determine exactly which variables produce the differences. And the second, to study whether significant differences exist in young people by relationship status – single or partnered – from the scores obtained in each of the dimensions in the autonomy questionnaire and the wellbeing questionnaire. Similarly, we also aimed to ascertain the variables that produce differences in each of the dimensions.

### Participants

The sample of young people was selected through an intentional non-probability sampling, consisting of 1,148 study subjects aged 16–21 (mean age = 18.20; *SD* = 1.8), 508 (44.3%) were Spanish (Madrid) and 640 (55.7%) Colombian (Bogotá); 60.1% (*n* = 690) were female and 39.7% (*n* = 456) male. Of the total sample, 38% (*n* = 436) were partnered and 61.5% (*n* = 706) were single. Of those who were partnered, 283 (64.9%) were female and 153 (35.1%) male. And in the young people who were single 57.5 (*n* = 406) were female and 42.5 (*n* = 300) male.

The study was conducted from late 2018 to early 2019. The participants were studying at universities and secondary schools. Data were also collected from young people who were employed, as well as from participants who were under the tutelage of child protection services. Young people who presented functional, physical or mental difficulties that made it impossible for them to participate in the study were excluded.

### Materials

#### Young People’s Assessment of Autonomy

Young people’s autonomy was measured using the Transition to Adulthood Autonomy Scale (EDATVA, Bernal et al., 2019). The scale comprises a total of 19 items organized into four dimensions: *self-organization* (six items, α = 0.80), *understanding context* (four items, α = 0.74), *critical thinking* (five items, α = 0.70) and *socio-political engagement* (four items, α = 0.77). Cronbach’s alpha for this set of items was 0.84 for the total sample. All the dimensions were measured using a four-point Likert scale, with 1 being strongly disagree and 4 being strongly agree.

#### Young People’s Assessment of Wellbeing

Young people’s wellbeing was assessed through the Spanish adaptation of Ryff’s Psychological Wellbeing Scale (Díaz et al., 2006). The scale is composed of 39 items organized in the following dimensions: *self-acceptance* (six items, α = 0.83), *positive relations with others* (six items, α = 0.81), *autonomy* (eight items, α = 0.73), *environmental mastery* (six items, α = 0.71), *personal growth* (seven items, α = 0.68) and *purpose in life* (six items, α = 0.83). There were a total of 17 inverse reagents amongst the items on the wellbeing scale (2, 4, 5, 8, 9, 13, 15, 20, 22, 25, 26, 27, 29, 30, 33, 34, and 36). Participants responded using a Likert-type scale format with scores ranging from 1 (strongly disagree) to 6 (strongly agree).

### Procedure and Data Analysis

In order to respond to the first research objective and to analyze whether there were differences by gender in the different dimensions of the autonomy and wellbeing questionnaires, and in line with Pardo and San Martín’s (2010) recommendations, we tested for assumption of normality and equality of variances (homoscedasticity). The former confirmed that the scores from each group constitute a random sample taken from the normal population and was calculated using the Kolmogorov-Smirnov and the Shapiro-Wilk tests, and Q-Q plots. We checked for homoscedasticity in the populations using Levene’s test (based on means). In the autonomy scale, although the equality of variances was met, thus ensuring that both groups (male and female) had equal variances, the same was not true of the assumption of normality given that the hypothesis that the sample came from a normal distribution was rejected in both groups (*ð* < 0.05 with both the Kolmogorov-Smirnov and the Shapiro-Wilk tests). The Q-Q plots also revealed that the values did not lie on the line, especially at the extremes, indicating that the theoretical distribution was not a good approximation of an empirical distribution, but an asymmetric distribution. Given the results, we then used the Mann–Whitney non-parametric *U*-test, which enabled us to analyze where the differences arose between males and females in the dimensions in the autonomy questionnaire. Subsequently, when the differences were identified, the variables that comprise the dimensions were analyzed individually in order to determine exactly which variables gave rise to gender differences. To this end, the Mann–Whitney *U*-test was again applied (the assumption of normality was not met). In the wellbeing scale, both assumptions were met (normality and homoscedasticity), given that the Q-Q plots clearly showed the absence of asymmetry and in Levene’s test the null hypothesis of equality of variances (*p* > 0.05) was met. Consequently, Student’s *t*-test was used for independent samples. This test was also used (the assumption of normality having been met) to study the gender differences in the variables that comprise the different dimensions that were significant in the comparison of scores between males and females.

The aim of the second objective was also to ascertain the differences in the dimensions of the autonomy and wellbeing questionnaires, but with regard to the variable relationship status – single or partnered. We proceeded in the same way as in the previous objective, starting with the verification of the assumption of normality and homoscedasticity. In the case of the autonomy questionnaire, we used non-parametric tests (Mann–Whitney *U*-test) given that the assumption of normality was again not met (neither in the total score of the dimensions nor in their variables). And in the case of the wellbeing questionnaire, given that the assumption of normality was met, we used parametric tests (Student’s *t*-test for independent samples). In order to determine the magnitude of the differences found between the different groups, the effect size was calculated using Cohen’s *d* statistic, for both the parametric and non-parametric tests. Despite the fact that the effect size was inconsistent and its interpretation confusing, we followed the recommendations made by Cohen (1992) given that there is no consensus regarding what magnitude of effect size is necessary to establish practical significance (Ferguson, 2009). Therefore, a value around 0.20 indicates a small effect, values around 0.50 a moderate effect and values around 0.80 and higher a large effect. As highlighted by Frías-Navarro et al. (2000), if we take into account that the value of the estimate of the effect size must be interpreted in the context of a study and specific area of research, a small effect size can be of great significance in certain fields.

As regards effect sizes, in most cases the magnitude of the differences was small. This is not surprising given that, as highlighted by Rosnow and Rosenthal (2009), effect sizes commonly found in the social sciences are often very small. Furthermore, as already mentioned, the estimate of the effect size must be interpreted in the context of a particular study and area of research (Frías-Navarro et al., 2000), in this case social sciences.

## Results

We start with the first objective of the study, to analyze the differences in the scores obtained in autonomy and wellbeing according to gender. In the autonomy scale, as explained in the procedure, the Mann–Whitney *U*-test and the Wilcoxon *W*-test and *Z-*value (a type of *U* and *W* statistic) were used. Table 1 shows that females present higher average scores than males in all the dimensions in the autonomy questionnaire. These differences range from 1.99 points in *socio-political engagement* to 42.79 points in *understanding context*. The significant difference is only in the latter dimension: *understanding context* (*p* < 0.05) in the female group given that this is the group that shows a higher average score (590.53 vs 547.74). However, the effect size is small (Cohen, 1992).

**TABLE 1:** Descriptive statistics and Mann–Whitney *U*-test for the dimensions in the autonomy questionnaire.

		*N*	Self-organization	Understanding context	Critical thinking	Socio-political engagement
Mid-range	Female	690	575.87	590.53	578.91	574.29
	Male	456	569.92	547.74	565.31	572.30
Mann–Whitney *U*-test			155687.500	145572.500	153585.000	156773.500
Wilcoxon *W* test			259883.500	249768.500	257781.000	260969.500
Z			−0.299	−2.164	−0.684	−0.100
*p*-value (bilateral)			0.765	0.030	0.494	0.920
Effect size (*d*)				0.127		

Taking a more detailed look at the analysis of this dimension, Table 2 shows which items are contributing to the main differences between both genders. It can be observed that in *understanding context*, females have higher average scores in the variables: *I defend my rights when I make important decisions* and *It’s important to express your ideas, even though your partner might get upset*. However, in the average range, males scored higher in the variable *If my rights are breached, I do everything to defend them* and *I use the available resources to denounce what I think is unfair*. We only found significant differences (*p* < 0.05) between males and females in the item: *It’s important to express your ideas, even though your partner might get upset*, with females presenting a higher average than males by more than 110 points. The magnitude of the effect size in this case is low-moderate (0.34).

**TABLE 2:** Descriptive statistics and Mann–Whitney *U*-test for the variables that constitute understanding context.

		*N*	EA49. I defend my rights when I make important decisions	EA56. If my rights are breached, I do everything to defend them	EA57. I use the available resources to denounce what I think is unfair	EA59. It’s important to express your ideas, even though your partner might get upset
Mid-range	Female	690	580.85	568.72	569.78	614.58
	Male	455	561.10	579.49	576.62	504.57
Mann–Whitney *U*-test			151559.500	154024.000	154873.500	125686.500
Wilcoxon *W* test			255299.500	392419.000	392578.500	228064.500
Z			−1.106	−0.579	−0.366	−6.335
*p*-value (bilateral)			0.269	0.563	0.715	0.000
Effect size (*d*)						0.343

For this same objective, the results for the wellbeing scale were analyzed using the *t*-test statistic for independent samples. Table 3 shows the descriptive data on the dimensions of the questionnaire, organized by gender, number of cases, mean and standard deviation (in the lower row in parentheses). In the table, it can be observed that the mean in *self-acceptance* is slightly higher in males (25,381) than in females (25,027), and the same is true of *environmental mastery* (25,449 vs. 25,284). However, in *positive relations with others*, the opposite occurs; it is higher in females (25,816) than males (25,018), and the same occurs with *autonomy* (34,318 vs. 33,996), *personal growth* (33,258 vs. 31,919), and *purpose in life* (27,109 vs. 26,993).

**TABLE 3:** Descriptive statistics and summary for the *t*-test for independent samples.

	Mean			Levene’s test for equality of variances		*t*-test for equality of means				
	Female	Male		*F*	Sig.	*t*	Degrees of freedom	Sig. (bilateral)	Difference in mean	Standard error of the mean
*N*	686	448								
Self-acceptance	25.027 (5.760)	25.381 (5.697)	Equality of variances met	0.035	0.852	−1.016	1132	0.310	−0.354	0.348
			Equality of variances not met			−1.018	962.959	0.309	−0.354	0.347
Positive relations with others	25.816 (6.297)	25.018 (5.873)	Equality of variances met	2.158	0.142	2.143	1132	0.032	0.798	0.372
			Equality of variances not met			2.175	1001.688	0.030 0.130	0.798	0.367
Autonomy	34.318 (6.810)	33.996 (6.835)	Equality of variances met	0.315	0.575	0.778	1132	0.437	0.322	0.414
			Equality of variances not met			0.777	953.100	0.437	0.322	0.414
Environmental mastery	25.284 (5.102)	25.449 (5.199)	Equal variances met	0.180	0.671	−0.526	1132	0.599	−0.164	0.312
			Equal variances not met			−0.524	942.672	0.600	−0.164	0.313
Personal growth	33.258 (5.225)	31.919 (5.185)	Equality of variances met	0.102	0.750	4.223	1128	0.000	1.338	0.316
			Equality of variances not met Effect size			4.230	958.884	0.000 0.137	1.338	0.316
Purpose in life	27.109 (5.803)	26.993 (5.624)	Equality of variances met	0.062	0.804	0.333	1132	0.739	0.116	0.348
			Equality of variances not met			0.335	976.656	0.737	0.116	0.345

Table 3 also shows the contrast of hypotheses of equality of variances based on Levene’s *F*-test. In all the dimensions the probability associated with Levene’s test (sig.) is greater than 0.05, therefore, the hypothesis of equality of variances is met. The variability is the same in both groups.

And lastly, Table 3 includes the *t*-test, the statistical significance, and the effect size (*d*) in those dimensions where significant differences between the groups were found. Assuming equal variances, it can be observed that there are significant differences between males and females in *positive relations with others* and *personal growth* (*p* < 0.05). In both cases, females scored higher on average (25,816 and 33,258, respectively) and effect sizes were small in both dimensions.

An in-depth analysis was performed with the two dimensions in which the differences between groups were significant. Specifically, as shown in Table 4, assuming equal variances there are significant differences in the variables: *I feel that I get a lot from my friendships* and *I know I can trust my friends, and they know they can trust me*, with females scoring higher (4.71 and 4.72, respectively). The effect size is low in both variables, but higher in the item: *I feel that I get a lot from my friendships* (*d* = 0.218).

**TABLE 4:** Summary of the *t*-test for the independent samples for positive relations with others.

	Media			Levene’s test for equality of variances		*t*-test for equality of means				
	Female	Male		*F*	Sig.	*t*	Degrees of freedom	Sig. (bilateral)	Difference in mean	Standard error of the mean
EB2. I often feel lonely because I have few close friends to share my problems with	4.29 (1.581)	4.40 (1.521)	Equality of variances met	2.099	0.148	−1.139	1126	0.255	−0.108	0.095
			Equality of variances not met			−1.149	967.049	0.251	−0.108	0.094
*N*	686	442								
I don’t have many people who want to listen to me when I need to talk	4.41 (1.534)	4.21 (1.573)	Equality of variances met	0.868	0.352	2.117	1125	0.234	0.200	0.095
			Equality of variances not met			2.106	926.657	0.235	0.200	0.095
N	684	443								
EB14. I feel that I get a lot from my friendships	4.71 (1.320)	4.42 (1.336)	Equality of variances met	0.660	0.417	3.543	1085	0.000	0.292	0.083
			Equality of variances not met Effect size	0.218		3.534	888.528	0.000	0.292	0.083
*N*	665	422								
EB20. It seems that other people have more friends than I do	3.78 (1.690)	3.96 (1.599)	Equality of variances met	8.708	0.003	−1.827	1124	0.068	−0.184	0.101
			Equality of variances not met			−1.848	985.879	0.065	−0.184	0.100
*N*	681	445								
EB26. I haven’t had many close and trusting relationships	4.13 (1.566)	3.94 (1.623)	Equality of variances met	2.787	.095	1.913	1124	0.056	0.185	0.097
			Equality of variances not met			1.898	922.258	0.058	0.185	0.098
*N*	682	444								
EB32. I know I can trust my friends, and they know they can trust me	4.72 (1.382)	4.51 (1.436)	Equality of variances met	1.051	0.306	2.437	1128	0.015	0.208	0.085
			Equality of variances not met			2.418 0.149	927.746	0.016	0.208	0.086
*N*	683	447								

Similarly, in this same dimension, although not statistically significant, a trend can be observed in which females obtain higher average scores than males, except in the items: *I often feel lonely because I have few close friends to share my problems with* and *It seems that other people have more friends than I do*, items in which males obtain higher average scores.

The same analysis was performed for *personal growth*. Table 5 shows that there are significant differences between male and female responses in the variables: *It’s been a long time since I stopped trying to make big improvements or changes in my life*; *I don’t want to try new ways of doing things; my life is fine as it is*, and *When I think about it, I haven’t really improved much as a person over the years*. Females score higher on average in all three items. The trend continues in the rest of the items in the dimension. The effect sizes are between low and moderate (*d* = 0.361, *d* = 0.149, and *d* = 0.161, respectively).

**TABLE 5:** Descriptive statistics and summary of the *t*-test for the independent samples for personal growth

	Media			Levene’s test for equality of variances		*t*-test for equality of means				
	Female	Male		*F*	Sig.	*t*	Degrees of freedom	Sig. (bilateral)	Difference in mean	Standard error of the mean
EB24. In general, over time I do feel that I’m still learning things about myself	5.15 (1.054)	5.04 (1.055)	Equality of variances met	0.368	0.544	1.702	1126	0.089	0.109	0.064
			Equality of variances not met			1.702	950.761	0.089	0.109	0.064
*N*	682	446								
EB30. It’s been a long time since I stopped trying to make big improvements or changes in my life	4.74 (1.393)	4.21 (1.573)	Equality of variances met	9.080	0.003	2.555	1127	0.011	0.223	0.087
			Equality of variances not met Effect size	0.361		2.518	906.437	0.012	0.223	0.089
*N*	682	447								
EB34. I don’t want to try new ways of doing things; my life is fine as it is	4.26 (1.770)	4.01 (1.524)	Equality of variances met	0.215	0.643	2.414	1125	0.016	0.247	0.102
			Equality of variances not met Effect size	0.149		2.489	1044.668	0.013	0.247	0.099
*N*	681	446								
EB35. I think it’s important to have new experiences that challenge what you think about yourself and the world	5.06 (1.118)	4.92 (1.201)	Equality of variances met	2.388	0.123	1.942	1128	0.052	0.136	0.070
			Equality of variances not met			1.914	904.962	0.056	0.136	0.071
*N*	683	447								
EB36. When I think about it, I haven’t really improved much as a person over the years	4.22 (1.588)	3.96 (1.651)	Equality of variances met	0.947	0.331	2.686	1125	0.007	0.264	0.098
			Equality of variances not met Effect size	0.161		2.665	928.165	0.008	0.264	0.099
*N*	680	447								
EB37. I have the feeling that over time I’ve developed a lot as a person	4.85 (1.193)	4.71 (1.244)	Equality of variances met	1.562	0.212	1.851	1123	0.064	0.137	0.074
			Equality of variances not met			1.835	920.753	0.067	0.137	0.075
*N*	680	445								
EB38. For me, life has been a continuous process of study, change and growth	5.07 (1.106)	4.81 (1.265)	Equality of variances met	11.474	0.001	3.582	1127	0.063	0.255	0.071
			Equality of variances not met			3.483	862.699	0.063	0.255	0.073
*N*	682	447								

The second research objective of the study, analyzing whether there are significant differences by relationship status – single or partnered young people – in the different dimensions of autonomy and wellbeing scales is explained below.

To answer this question, the Mann–Whitney *U*-test was performed on the autonomy scale, the results of which are shown in Table 6. It can be observed that there are no significant differences between single and partnered young people in any of the dimensions in the autonomy questionnaire (*p* > 0.05). However, descriptively it can be observed that in *self-organization* and *critical thinking* partnered young people score higher on average, while single young people score higher in *understanding context* and *socio-political engagement.*

**TABLE 6:** Descriptive statistics and Mann–Whitney *U*-test for the dimensions of autonomy.

		*N*	Self-organization	Understanding context	Critical thinking	Socio-political engagement
Mid-range	Partnered	436	578.75	568.22	577.35	562.11
	Single	706	568.65	575.13	569.51	578.90
Mann–Whitney *U*-test			151621.000	152478.500	152230.000	149816.000
Wilcoxon W test			402607.000	247744.500	403216.000	245082.000
Z			−0.504	−0.347	−0.391	−0.839
*p*-value (bilateral)			0.614	0.728	0.695	0.402

In relation to the results obtained in the wellbeing questionnaire, in order to comply with the assumption of normality we used Student’s *t*-test for independent samples. Table 7 shows significant differences between single and partnered young people in *environmental mastery* and *purpose in life*, with higher average scores for partnered young people. The effect size on the dimensions was *d* = 0.132 and *d* = 0.145, respectively. While the differences are not significant, the same trend can be seen in *self-acceptance*, *autonomy* and *personal growth*, where partnered young people also obtained a higher average score. Only in *positive relations with others* did single young people obtain higher scores.

**TABLE 7:** Descriptive statistics and summary of the *t*-test for independent samples for the psychological wellbeing questionnaire.

	Media			Levene’s test for equality of variances		*t*-test for equality of means				
	In a relationship	Not in a relationship		*F*	Sig.	*t*	Degrees of freedom	Sig. (bilateral)	Difference in mean	Standard error of the mean
*N*	431	702								
Self-acceptance	25.218 (5.643)	25.105 (5.791)	Equality of variances met	0.629	0.428	0.321	1131	0.748	0.112	0.350
			Equality of variances not met			0.323	927.945	0.747	0.112	0.348
Positive relations with others	25.273 (6.218)	25.626 (6.089)	Equality of variances met	0.009	0.923	-0.940	1131	0.348	−0.353	0.375
			Equality of variances not met			−0.935	895.066	0.350	−0.353	0.377
Autonomy	34.320 (6.784)	34.114 (6.853)	Equality of variances met	0.079	0.778	0.494	1131	0.622	0.206	0.417
			Equality of variances not met			0.495	916.911	0.621	0.206	0.416
Environmental mastery	25.758 (5.058)	25.078 (5.185)	Equality of variances met	0.032	0.858	2.164	1131	0.031	0.680	0.314
			Equality of variances not met Effect size	0.132		2.177	927.116	0.030	0.680	0.312
Personal growth	32.841 (5.058)	32.642 (5.261)	Equality of variances met	0.000	0.984	0.619	1127	0.536	0.199	0.322
			Equality of variances not met			0.619	906.655	0.536	0.199	0.322
Purpose in life	27.568 (5.481)	26.736 (5.869)	Equality of variances met	2.549	0.111	2.375	1131	0.018	0.831	0.350
			Equality of variances not met Effect size	0.145		2.414	957.433	0.016	0.831	0.344

Following the same procedure as with the gender variable, we analyzed the items in the different dimensions on the wellbeing scale where significant differences were found, which revealed differences between single and partnered young people. Table 8 shows significant differences in *environmental mastery* between single and partnered young people in the variable *The demands of everyday life often get me down*, where partnered young people obtain higher scores (4.11) than single young people (3.91). This gives rise to an effect size of *d* = 0.136. Without showing significant differences, this trend is maintained in all items except one: *I’m quite good at handling most of my daily responsibilities*, in which single young people obtain a higher average (4.41 vs. 3.91).

**TABLE 8:** Descriptive statistics and summary of the *t*-test for independent samples for environment mastery.

	Media			Levene’s test for equality of variances		*t*-test for equality of means				
	Partnered	Single		*F*	Sig.	*T*	Degrees of freedom	Sig. (bilateral)	Difference in mean	Standard error of the mean
EB5. I find it difficult to direct my life toward a path that satisfies me	4.14 (1.564)	3.99 (1.581)	Equality of variances met	0.001	0.975	1.539	1119	0.124	0.149	0.097
			Equality of variances not met			1.543	906.239	0.123	0.149	0.097
*N*	426	695								
B11. I’ve been able to build a home and a way of life to my liking	3.91 (1.477)	3.82 (1.544)	Equality of variances met	1.432	0.232	1.013	1100	0.311	0.095	0.094
			Equality of variances not met			1.024	930.801	0.306	0.095	0.093
*N*	425	677								
EB16. In general, I feel that I’m responsible for my situation in life; it’s fine as it is	4.57 (1.228)	4.50 (1.376)	Equality of variances met	7.680	0.006	0.874	1125	0.382	0.071	0.081
			Equality of variances not met			0.898	986.273	0.370	0.071	0.079
*N*	427	692								
EB22. The demands of everyday life often get me down	4.11 (1.451)	3.91 (1.467)	Equality of variances met	0.001	0.969	2.304	1117	0.021	0.207	0.090
			Equality of variances not met Effect size	0.136		2.310	909.785	0.021	0.207	0.090
*N*	427	692								
EB28. I’m quite good at handling most of my daily responsibilities	4.22 (1.182)	4.41 (1.247)	Equality of variances met	0.353	0.553	0.228	1121	0.820	0.017	0.075
			Equality of variances not met			0.231	941.157	0.817	0.017	0.074
*N*	428	695								
EB39. If I were unhappy with my situation in life, I’d take the most effective steps to change it	4.83 (1.345)	4.81 (1.325)	Equality of variances met	0.019	0.889	0.235	1121	0.815	0.019	0.082
			Equality of variances not met			0.234	890.517	0.815	0.019	0.082
*N*	427	696								

Table 9 shows there are significant differences in *purpose in life* between single and partnered young people in the variables: *I enjoy making plans for the future and working to make them a reality* and *I have a clear direction and purpose in my life*, where partnered young people score the highest (5.07 and 4.67, respectively) with an effect size of *d* = 0.139 and *d* = 0.149, respectively. Again, this is a trend that is observed in the rest of the items in this dimension with the exception of *I’m an active person, I carrying out the projects I set myself*, in which single young people obtain a higher average.

**TABLE 9:** Descriptive statistics and summary of the *t*-test for independent samples for purpose in life.

	Media			Levene’s test for equality of variances		*t*-test for equality of means				
	Partnered	Single		*F*	Sig.	*t*	Degrees of freedom	Sig. (bilateral)	Difference in mean	Standard error of the mean
EB6. I enjoy making plans for the future and working to make them a reality	5.07 (1.094)	4.91 (1.173)	Equality of variances met	1.892	0.169	2.256	1124	0.024	0.158	0.070
			Equality of variances not met			2.294	951.661	0.022	0.158	0.069
			Effect size	0.139						
*N*	428	698								
EB12. I’m an active person, I carrying out the projects I set myself	4.57 (1.204)	4.61 (1.216)	Equality of variances met	0.195	0.659	−0.549	1116	0.583	−0.041	0.075
			Equality of variances not met			−0.551	911.833	0.582	−0.041	0.074
N	428	690								
EB17. I feel good when I think about what I’ve done in the past and what I can do in the future	4.43 (1.261)	4.32 (1.340)	Equality of variances met	0.456	0.500	1.332	1122	0.183	0.107	0.080
			Equality of variances not met			1.351	945.747	0.177	0.107	0.079
*N*	428	696								
EB18. My goals in life have been more a source of satisfaction than frustration to me	4.45 (1.271)	4.34 (1.316)	Equality of variances met	1.071	0.301	1.342	1120	0.180	0.107	0.080
			Equality of variances not met			1.353	925.952	0.176	0.107	0.079
*N*	427	695								
EB23. I have a clear direction and purpose in my life	4.67 (1.330)	4.46 (1.449)	Equality of variances met	5.452	0.020	2.504	1123	0.012	0.216	0.086
			Equality of variances not met			2.555	965.250	0.011	0.216	0.084
			Effect size	0.149						
*N*	429	696								
EB29. I don’t know what I want to achieve in life	4.58 (1.521)	4.35 (1.617)	Equality of variances met	4.242	0.040	2.384	1122	0.117	0.232	0.097
			Equality of variances not met			2.419	943.071	0.116	0.232	0.096
*N*	425	697								

## Discussion

The results for the first objective, which aimed to analyze whether there were differences in the autonomy scale and the wellbeing scale by gender, show that in the autonomy scale the female sample give higher average scores than the male sample in all the dimensions in the questionnaire, with a significant difference in *understanding context*. The items that show significant differences within this dimension indicate that females tend to defend their rights to a greater extent when making important decisions and consider it important to express their opinions, even if they are contrary to their partner’s opinions. This implies that young females are less dependent on their partners in decision making given that their evaluation of themselves helps to build and improve their own self-esteem (Didonato and Krueger, 2010). They also analyze their opinions in relation to contexts with greater autonomy. *Understanding context* is based on a greater understanding of the situations that affect them by establishing an attitude toward decision making from a personal, social and political perspective via the search for more information relating to their rights and that which helps them to form and express their own opinions.

In terms of wellbeing, the male sample obtained higher average scores in the dimensions of *self-acceptance*, and *environmental mastery*, while the female sample scored higher in the rest of the dimensions and significantly higher in *positive relations with others* and *personal growth*. In the study performed by Sun et al. (2016), with a sample of 277 adolescents in Hong Kong aged 12–16, their findings determined greater *self-acceptance* and *autonomy* in adolescent males, and the same is true of the study performed by Mayordomo et al. (2016) with a sample of young people (*N* = 246; ≥18 years, *M* = 23.6). Melo and Mota (2013), in their research with a sample of 827 young people aged 13–25, determined that males score higher in relation to self-esteem (Antunes and Correia, 2016). However, with respect to *environmental mastery*, the study by Mayordomo et al. (2016) reports similar scores between males and females.

In terms of *positive relations with others*, the female sample felt that their friendships made an important difference to their lives and perceived that they had mutually trusting relationships with friends (significant differences were found with respect to males). On the other hand, the male sample felt more alone or had fewer friends and considered that other people had more friendships than they did, although these differences were not significant. Similar findings were made in the study by Sun et al. (2016), in which adolescent males experienced isolation more often, while, to a greater extent, strong relations with others were part of adolescent females’ identities. In addition, in all the items that comprise *personal growth*, females score higher, with significant differences to males in the variables: *It’s been a long time since I stopped trying to make big improvements or changes in my life*, *I don’t want to try new ways of doing things; my life is fine as it is*; and *When I think about it, I really haven’t improved much as a person over the years*. Similar findings were also made in the study by Mayordomo et al. (2016) in *positive relations with others* and *personal growth*, as well as in the study by Steca et al. (2002) with young females obtaining the highest scores.

The finding of the second objective, which aimed to analyze whether there are differences in the dimensions of the autonomy scale and the wellbeing scale by relationship status, show that there are no significant differences between single and partnered young people in any of the dimensions in the autonomy questionnaire. However, there is a descriptive tendency in *self-organization* and *critical thinking* of partnered young people obtaining higher scores. In this regard, the study by Melo and Mota (2013) found that young people whose parents had separated became more sensitive, withdrawn and defensive when it came to developing or establishing romantic relationships. As a result, s*elf-organization* and *critical thinking* can be influenced by this precept in young people who have this family background. The authors also argue that the same is true of self-esteem. This predictive indicator of young people’s psychological wellbeing influences both the type and quality of love. Self-esteem is directly linked not only to the acceptance of oneself, but also to the acceptance of how others see you (Rocha, 2008). However, with respect to *understanding context* and in *socio-political engagement*, single young people obtain higher scores. In contrast, according to the study by Monteiro et al. (2006) ambivalence toward or confidence in the romantic partner are positive predictor variables of sociability and social involvement. Young people who have higher levels of self-confidence tend to trust others more in society and express a greater predisposition to socialize (Matos et al., 2001). They also highlight that the avoidance of love negatively predicts social participation, given that evasive people are more focused on themselves, which decreases their availability when it comes to establishing relationships (Melo and Mota, 2013).

In terms of the wellbeing scale, significant differences were found between single and partnered young people in *environmental mastery* and *purpose in life*, with higher average scores in partnered young people. In *environmental mastery*, significant differences were found in the variable *The demands of everyday life often get me down*, with higher scores obtained by partnered young people. This descriptive tendency is maintained in the rest of the items in this dimension with the exception of *I’m quite good at handling most of my daily responsibilities*. In turn, Melo and Mota (2013) argue that self-esteem is a predictive indicator of psychological wellbeing, especially in young people who recognize the quality of love, and those who contemplate seeking a romantic partner. In the same study, ambivalence toward or confidence in the romantic partner positively predicts happiness, in other words, the perception of happiness increases according to the level of confidence one has in one’s partner. Similarly, in the study by Ramalho (2008), with a sample of 200 young people aged 18–25, romantic relationships based on anxiety and insecurity have a negative influence on psychological wellbeing. Similar results were found in the study by Rivera et al. (2011) involving 120 young people aged 18–26, whose results indicate that satisfaction in the relationship decreases according to levels of anxiety and fear of intimacy.

The item *I’m quite good at handling most of my daily responsibilities*, gave higher scores in single young people, given that they consider themselves more capable than partnered young people at handling the responsibilities of their daily lives. In a study by Matos et al. (2001) with a sample of 365 young people with an average age of 15.5, they found young people had higher levels of dependency on peers and romantic partners. Taking into account that this is a developmental stage in which young people evaluate themselves, dependency can be understood as seeking proximity to peers and acceptance from others. Single young people are less dependent and assume their everyday responsibilities more easily. In terms of *purpose in life*, partnered young people enjoy making plans for the future and attempting to undertake those plans significantly more than single young people, they also have a clearer idea about their life goals. This descriptive tendency is maintained in the rest of the items in this dimension with the exception of *I am an active person in carrying out the projects that I proposed for myself*. Single young people consider themselves more active when executing the projects they propose, given that they have a more individualized vision and are less dependent on establishing affective bonds with others. However, they do consider themselves more diligent when addressing their purpose and projects in life without necessarily enjoying making future plans (Matos et al., 2001).

In short, the findings in this study show that with respect to the gender variable there are differences in the dimensions of *positive relations with others* and *personal growth* and *understanding context*, with females obtaining the highest scores. As for the relationship status variable, differences were found in *environmental mastery* and *purpose in life*, with higher scores obtained by partnered young people. However, no differences were found in the different dimensions in the autonomy questionnaire between single and partnered young people.

A possible limitation in this study could be seen as the differences in sample sizes between females (690) and males (456), and partnered (436) and single (708). However, we believe that these differences do not significantly bias the results given that both the gender and the relationship status samples are very large. Nevertheless, to check that the differences between the sample sizes were not altering the results, the comparison analyses were replicated by equalizing the sample sizes (male and female = 456; single and partnered = 436). The differences found between the samples were the same as those obtained in the analyses performed with the different sized samples. On a final note, we believe it would be interesting to perform a more in-depth study on the level of autonomy and psychological wellbeing in emerging adults taking into account gender and relationship status in relation to family structure. In other words, how these two variables might influence autonomy and wellbeing depending on family status, i.e., whether parents are separated, divorced, etc.

## Data Availability Statement

All datasets generated for this study are included in the article/supplementary material.

## Ethics Statement

This study was performed in accordance with the recommendations of the Ethics Committee of the Universidad Santo Tomás (Bogotá, Colombia) and the Ethics Committee of the Universidad Nacional de Educación a Distancia (Madrid, Spain) and is, therefore, in accordance with the Declaration of Helsinki (seventh revision 2013, Fortaleza, Brazil). All participants were given a full description of the study and informed that participation was voluntary. Informed consent was obtained. In the case of minors, in addition to their consent, consent from parents or guardians was also obtained. Written informed consent to participate in this study was provided by the participants’ legal guardian/next of kin.

## Author Contributions

FG-C led the project. DA created the database, performed the statistical analyses, wrote the methodological section of the article, and the methodological limitations of the research. DA and GC wrote the results section. FG-C and IM-S wrote the introduction and the theoretical framework, reviewed the references, and obtained financial support for the study. FG-C, IM-S, GC, and DA prepared the discussion section and reviewed the first draft of the article. All authors contributed to the article and approved the submitted version.

## Conflict of Interest

The authors declare that the research was conducted in the absence of any commercial or financial relationships that could be construed as a potential conflict of interest.
